# Structuring the situation: Organizational goals trigger and direct decision-making

**DOI:** 10.3389/fpsyg.2023.1140408

**Published:** 2023-03-29

**Authors:** Henrich R. Greve

**Affiliations:** Entrepreneurship and Family Enterprise, INSEAD, Singapore, Singapore

**Keywords:** goals, Carnegie School, decision-making, organizations, problemistic search

## Abstract

Organizational goals are assigned to individuals, and thus differ from goals that individuals voluntarily adopt. The Carnegie School has a significant research stream on how organizations are affected by goals, with a focus on how disappointing performance disrupts regular organizational behavior and triggers a search for alternative actions. We have a good understanding of the organization-level process of setting aspiration levels, triggering search for alternatives, and making decisions, but the individual-level mechanisms contributing to it are less well known. An assessment of the progress of Carnegie School research so far reveals a list of research questions that should be resolved in order to understand how individual updating of aspiration levels, triggering of search, directing of search, and decision-making help explain organizational responses to goals. The role of construal, or interpretation, in guiding these processes is a central theoretical mechanism that needs further investigation.

## Introduction

Study of goals in psychology is a rich theoretical and empirical enterprise. Much of it has been oriented toward goals that are personalized in the sense of either explicitly referring to personal goals or implicitly assuming some level of personal control over goal adoption and pursuit (e.g., Austin and Vancouver, 1996; Brandstätter and Bernecker, 2022). This has led to productive research streams on issues like goal selection (Heckhausen et al., 2010), goal pursuit (Locke and Latham, 2002; Richter et al., 2016), goal attention (Dijksterhuis and Aarts, 2010), and goal persistence (Brandstätter and Bernecker, 2022). In combination, these research streams produce a comprehensive view on how individuals select personal goals, pursue them, and either persist in this pursuit or instead disengage and pursue alternative goals (Brandstätter and Bernecker, 2022). Goals are also found in the psychology of decision-making under risk (Kahneman and Tversky, 1979; Heath et al., 1999).

The personalized view of goals is in striking contrast to how goals in organizations are seen by practicing managers and researchers. Although the conceptualization and use of goals is often blended, three branches can be identified. First, goals control people in regular organizational behavior through their connection to managerial evaluation and incentives. This effect of goals has been treated in the economic literature on incentives (Lazear, 2000) and the management literature on rewards (Ethiraj and Levinthal, 2009; Lee et al., 2018). Second, goals interfere in regular organizational behavior and trigger search for alternative courses of action (Greve, 2003b; Posen et al., 2018). This effect has been treated in the literature on organizational responses to performance feedback (Audia and Greve, 2021; Kotiloglu et al., 2021). Third, goals direct the search for alternative courses of actions by allowing interpretation of the problem (March and Simon, 1958; March and Olsen, 1975). This effect is yet to be fully explored because it requires examination of organizational responses to multiple goals, which has seen little examination so far (but see Audia and Brion, 2007; Greve, 2008; Gaba and Greve, 2019; Sobrepere et al., 2022).

In management practice, assigned goals are common and often used in combination (often denoted Key Performance Indicators). A popular practical application is the use of multiple goals that allow managers to monitor the performance of their organizational unit and its individual subordinates and direct attention to those who appear to be having low performance for review and improvement, and to those who appear to be having high performance for reward and promotion. This use of goals is taught in required MBA courses and embedded in managerial practice. It belies the idea of personalized goals chosen by the individual.

As a result, there is some mismatch between the use of goals in organizations and psychological research on goals. How goals control and regulate people has seen significant research in goal-setting theory (Locke and Latham, 1990, 2002) and the economics of incentives (e.g., Lazear, 2000), and is largely seen as a solved problem. Accordingly, current work in organizational theory is less interested in the controlling effect of goals than in how goal shortfalls interrupt regular organizational life and lead to search and decision-making that change organizational behaviors (Posen et al., 2018; Audia and Greve, 2021). Understanding when and how goals trigger change is an unsolved problem, as has been pointed out in recent articles discussing how much needs to be learned in order to fully understand organizational responses to low organizational performance (Posen et al., 2018; Audia and Greve, 2021) and their consequences for organizational strategy (Greve, 2021). To address this problem, the Carnegie School literature makes important theoretical distinctions with empirical import (Cyert and March, 1963). It differentiates between the goal dimension—what is the goal about—and the aspiration level—what is the performance level below which search for alternative behaviors may be initiated. It specifies a sequence in which performance on the goal variable below the aspiration level starts search for alternatives followed by a decision on whether each alternative should be implemented. Because the decision-makers seek to satisfice—find behaviors estimated to give performance above the aspiration level—alternatives are evaluated sequentially, and the search stops once a sufficiently promising has been found (Cyert and March, 1963).

How can we combine the concerns of psychology and organization theory and move toward a more integrated line of research? This paper takes three steps. First, it outlines major theoretical and empirical ideas of organizational theory and goal setting with a focus on insights from the Carnegie School, which is the pioneering and currently leading stream of research on organizational goals. Next, it discusses how research in the Carnegie School has revealed important gaps in our knowledge of individual responses to organizational goals. Finally, these point to areas of research in which Carnegie School research and psychology research have important complementarities, and to novel questions in psychology that derive naturally from the Carnegie School research on organizational goals. The goal is to invite a conversation of theory and evidence between two fields of research, each with expertise required to address this theoretical agenda and with obvious complementarities in knowledge foundation, theoretical approach, and empirical procedures.

A central feature of this discussion is that the common observation that organization theory and psychology produce theory and evidence at different levels of analysis, while correct, is not the main reason for the current mismatch of these two branches of goal research. Instead, the main reason is that organizational *use* of goals has consequences for the decision-making of their employees, which, in turn, *produces* organizational change. In this view of goals, the different levels of analysis are not problematic. The organization structures the situation faced by the individual. The individual behavior is oriented toward organizational goals and organizational actions, and the question to answer is what behaviors are produced.

## Goals in the Carnegie School

The main origin of thinking about goals in organizational theory is the Carnegie School, which developed the idea of the organization as having sets of goals that can be independent, hierarchically organized, causally linked, or some combination of these (Simon, 1947; March and Simon, 1958; Cyert and March, 1963). Organizational decision-making is boundedly rational, meaning that the decision-maker seeks to choose alternatives that have expected beneficial outcomes but has limited capability to interpret the situation, construct alternatives, tally their possible consequences and associated likelihoods, and integrate this information (Simon, 1962). Because all these actions need to be executed to be fully rational, limits on the decision-maker capabilities place boundaries on the achievable degree of rationality, hence leading to boundedly rational decision-making.

The boundedly rational decision-maker is dependent on goals for the following reasons:

1. Goals allow satisficing through comparison of performance and aspiration levels, thus identifying areas of activity in which outcomes are good enough, so decisions are not needed. This reserves attention for areas in which search for alternatives and subsequent decision-making may be needed (Cyert and March, 1963).
2. Goals are turned into numeric specifications, so they can be tracked through accounting systems, and decision-makers can compare the performance with adaptive aspiration levels set through observation of peers or historical performance (Cyert and March, 1963). The same comparison can be used to guess whether an alternative is good enough to be adopted, and hence allows satisficing by stopping the search for additional alternatives.
3. Goals allow localization of the search for alternatives, as many (but not all) goals are indicative of what organizational activities are currently problematic and hence could be targeted for change (Cyert and March, 1963).

Through these three features, goals conserve the energy and direct the attention of the boundedly rational decision-maker by reducing the number of decision-making occasions, the scope of decisions, and the alternatives considered.

Just as organizations have structures, so do the goals defined by organizations and assigned to individuals. The effect of organizational goal structures depends on the degree to which the goals are designed for optimal organizational responses. An apparent minimum degree of design is to have multiple goals that each indicate some set of organizational activity, for example those belonging to a function or division of the organization, and those are treated separately (Cyert and March, 1963). Research using this conceptualization has shown it to hold for broad goals such as profitability, safety, R&D progress, and alliances (Baum et al., 2005; Baum and Dahlin, 2007; Shipilov et al., 2011; Gaba and Greve, 2019). A higher degree of design is to have multiple goals that are hierarchically organized because the lower-level goals are thought to be causal in producing the higher-level goals (March and Simon, 1958). Research using this conceptualization has also shown some support (Gaba and Joseph, 2013; Mazzelli et al., 2019; Sobrepere et al., 2022). There is also significant research on a third and less designed goal structure in which an overarching goal such as profitability acts as a “master switch” to trigger changes across a broad range of activities (Greve, 2003b). Research using this conceptualization has very substantial support, showing that the organization-wide goal of profitability triggers changes across a wide range of organizational behaviors (see Shinkle, 2012; Kotiloglu et al., 2021).

Connecting these goal conceptualizations and their effects to individual action requires going through the details of the process, and this approach also helps identify gaps in the theory that require further attention. Let us start with a summary of the theoretical assumptions. First, although goals are commonly thought to be “organizational,” each goal is assigned to an organizational unit, a manager leading the unit, or a specific person in the unit. Organizational goals are personalized through assignment to individuals, which is not quite analogous to personalized goals in psychology because personal commitment may be lacking. Incentives are commonly used to produce the same effect as personal commitment.

Second, goals are specified as numerical items in some accounting system and performance on the goal dimension is assessed periodically. An aspiration level for each numerical goal variable is held by the individual, and is updated through comparison with past performance outcomes and peer performance outcomes (Cyert and March, 1963; Lant, 1992; Blettner et al., 2015). Aspiration levels may also be explicitly specified by the organization, but as goal commitment research shows, ultimately the individual’s commitment determines the actual aspiration level (Locke et al., 1988). The satisficing heuristic means that performance above the aspiration level is “good enough” and thus seen as insufficient reason to search for improvements in organizational behavior (Cyert and March, 1963; Levinthal and March, 1993).

Third, failure of performance to meet the aspiration level implies search for a solution, usually in the form of changed behaviors rather than greater effort. Organizations are typically operated at high workloads, so interruptions in the form of search for solutions can produce unpredictable and large delays in both regular work and the search progress (Glynn et al., 2019). Because performance on organizational goals results from routines involving multiple people and associated production assets, reorganizing the routines, replacing the individuals, and replacing technologies or assets are common solutions that decision-makers will search for (Gavetti et al., 2012; Shinkle, 2012).

### Satisficing

The foundation of the satisficing heuristic in organizational decision-making was decision-making triggered by an environmental stimulus and resolved through finding an alternative estimated to meet or exceed the minimally satisfactory threshold on all relevant criteria (March and Simon, 1958). This theory was later extended to define performance below the aspiration level on an organizational goal as an internal stimulus that leads to search for alternatives (known as problemistic search) (Cyert and March, 1963). The theory assumes that multiple goals are in operation, and whatever part of the organization is responsible for one specific goal will devote time and resources to searching for alternative behaviors if performance falls below the aspiration level. Goals are independent, aspiration levels are adaptive through historical and peer comparison, and search follows a heuristic of starting near the performance shortfall and current behaviors before spiraling outward if no satisficing alternative can be found (Cyert and March, 1963).

The bulk of evidence testing this model examines the goal of firm profitability, usually operationalized as return on assets, and looks at a broad range of behaviors that are substantial enough to be viewed as solutions. Examples include increasing research and development expenditures (Greve, 2003a; Rudy and Johnson, 2016), new product launch or update (Giachetti and Lampel, 2010; Gaba and Joseph, 2013), investment in assets (Audia and Greve, 2006; Arrfelt et al., 2013), change of strategy (Schimmer and Brauer, 2012; Kolev and McNamara, 2022), and change in alliances (Shipilov et al., 2011; Lungeanu et al., 2016).

The total evidence is impressive, well in excess of 200 studies, but it also has unclear foundation in psychological processes and unclear implications for psychological research. Behaviors such as these result from search triggered either by the CEO as an individual or the top management team, the details are developed elsewhere in the organization, the search is likely to end with a choice among alternatives, and the approval of the final action is done by the top management team or board of directors. These elaborate processes are organizational in nature (Levinthal and March, 1993; Levinthal and Rerup, 2021), though individuals like the CEO or groups like the board of directors intervene with great impact.

Empirically, this research is very successful in documenting satisficing behavior with respect to the goal of profitability and an adaptive aspiration level. Theoretically, it leaves gaps open for further exploration. The first gap is that examination of an organizational profitability goal differs from the theory on organizations having multiple goals assigned to organizational units and decision-makers, with responses matching the goal showing performance below the aspiration level. There are studies showing such effects of specific goals, however, and these indicate a way forward. For example, market share below the aspiration level leads to changed market position (Greve, 1998), and accidents lead to improved safety (Baum and Dahlin, 2007; Madsen and Desai, 2010). The theoretical assumption that multiple goals exist and are addressed separately appears to be valid.

A second gap lies in documenting that aspiration levels are adaptive also for other goals than profitability. Firm profitability is a special goal that invites both tracking of past performance and comparison with peers because those are exactly the kind of comparisons that outsiders, especially equity analysts and investors, will make. Many internal goals do not have readily available peers, though some, such as division-level profitability measures, do. What we know about other types of goals should be an invitation for further examination. Mutual funds managers have goals that are readily comparable over time and across peers, but the career implications of their performance relative to aspiration levels lead them to react differently from how firms react to profitability (Kacperczyk et al., 2015). Sports teams also have goals that are readily comparable, but these goals occur in a tournament context, which also alters responses (Moliterno et al., 2014). Similar responses are also seen for individuals engaged in tournaments (Boyle and Shapira, 2012). Whether such effects result from different adjustments of aspiration levels or different reactions to performance needs further examination, and extension to goals that are not readily comparable is a natural next step.

A third gap lies in documenting the process underlying aspiration-level updating and satisficing. Evidence that employees satisfice on organizationally assigned goals is currently scarce (but see Kacperczyk et al., 2015; Greve et al., 2019b). Such evidence is needed to know whether the observed satisficing is individually determined or enforced by organizational processes such as periodical performance reporting and reviews, which are often connected with direct incentives like pay and indirect incentives such as promotions or role expansions. A potential objection to the proposal that aspiration-level updating weighs peer comparison and own past performance is the mental effort of this operation, suggesting that this updating rule may not be a good prediction of individual updating. While this may be true, there is evidence of individuals engaging in goal-oriented behavior and complex mental accounting using performance feedback (Billinger et al., 2021; Bergenholtz et al., 2023) even without conscious deliberation (Dijksterhuis and Aarts, 2010), suggesting that individuals can be sophisticated. Clearly there is a tension between the sophisticated individual and the organization seeking to control aspiration levels through specifying numeric goals, and this needs to be explored further.

### Numeric control

Organizations make extensive use of numeric specifications to measure progress toward goals. This practice has long been known to be problematic for goals that are ambiguous, qualitative, or multi-dimensional, including such seemingly simple goals as a healthy work environment, high-quality products, and good R&D progress. Typically giving goals numeric specifications cause decision-maker attention to collapse from the broader intended goal to the narrower measured goal (Kerr, 1975). Organizations still use this simplification because it fits accounting processes geared toward numeric outputs, enables management to impose stricter incentives, and is coupled with a general “magic numbers” belief that anything that truly matters should be measurable (March, 1996).

This view of organizations being primarily guided by numeric performance compared with aspiration levels aligns with much of what we know about organizations, but it is also narrow. The primary problem is that decision-makers may combine an emphasis on the numeric performance measure with awareness of the broader goals. Thus, the numeric specification of the goal triggers search, but an initial step in the search may be examination of how well the organization does on dimensions of the goal that are not readily measurable. Such broader assessment of the situation can direct the search for solutions in ways that current theory does not capture.

### Directing search

The model of search specified by Cyert and March (1963) was, in their words, simple-minded, taking as a first step proximity to the problem (the goal variable) and the solution (current behaviors), followed by broad search or search in vulnerable areas of the organization if no satisficing solutions were found in the proximate search. Although this is a good initial description of how organizations search with some evidence in favor (Iyer et al., 2019), there are theoretical and empirical reasons to submit this model of search to closer scrutiny (Greve, 2018). We should start by noting that the evidence on search in response to focused goals such as low market share and low safety offers support to the simple-minded search model (Greve, 1998; Baum and Dahlin, 2007; Madsen, 2009), though it is support of the simplest kind because the match of problem and solution is so obvious.

Apart from the literature on responses to focused goals, the evidence on simple-minded search is remarkably limited. Many studies show that organizational search is more often local than distant (Laursen, 2012; Posen et al., 2018), but this support is weakened by the fact that most studies investigate organizational search in general rather than problemistic search specifically. Organizations also do routine search such as R&D (Cyert and March, 1963). The support is weakened even more by the common finding that local search tends to be more efficient than distant search, both during the routine R&D process and during problemistic search (Knudsen and Levinthal, 2007; Laursen, 2012), so organizations may favor local search simply because it is the best form of search.

Problemistic search may still be different because decision-makers can distinguish between problems that have proximate solutions and problems that require more distant search. As an example of the former, low profitability is addressed through resource conservation when the firm holds little financial resources, unlike when its financial reserves are great (Kuusela et al., 2017). As an example of the latter, a biopharmaceutical firm having fewer new product introductions than its aspiration level will not have innovative products ready for launch, and must instead take the long route of increasing R&D expenditures and R&D alliances (Tyler and Caner, 2016). Indeed, slow progress in R&D leads pharmaceutical firms to move from local to distant search (Hoang and Ener, 2015), just as the simple-minded model of search predicts. Early findings thus favor the model of simple-minded search, but more evidence is needed for a conclusive answer.

Answering this question requires consideration of the decision-maker construal of the situation. Construal processes are central in the Carnegie School (see March and Olsen, 1975), just as they are central in social psychology (Ross and Nisbett, 1991; Wilson, 2022),^1^ but theoretical and empirical work has relied on the concept of simple-minded search to such an extent that less attention has been devoted to construal. This theoretical stance has resulted in studies that were not designed to examine the construal process and its effects, so we are currently left seeking to draw implications from studies that had different goals.

One path toward understanding construal processes in organizations is to examine whether decision-makers integrate information from multiple goals and use it to direct search and make decisions. Recent work has yielded suggestive findings. There is good evidence that profitability is a goal that typically takes precedence over other goals (e.g., Greve, 2008; Smulowitz et al., 2020), so one might expect an airline with low profitability and a fleet with weak safety record not to make the aircraft purchases necessary to obtain a safer fleet. In fact, the opposite is true, possibly indicating that low profitability and high risk of accident is construed as a situation that threatens the existence of the airline (Gaba and Greve, 2019). Firms under siege for weak governance practices sorted themselves into low-profitability firms resisting efforts to improve governance, higher-profitability firms improving governance, and good-governance firm improving governance even further (Rowley et al., 2017). The findings are suggestive of construal directing organizational search, as this sorting suggests that governance was seen as a distraction, a shortcoming, or an advantage, depending on the configuration of profitability and governance in each firm. This conclusion is merely suggestive, however, as it derives from interpretations of firm reactions.

Clearer evidence can be drawn from analyses of sports, which often have simpler decision structures. Football team fourth-down plays suggest that teams were switching between viewing the situation as a short-term problem of continuing the drive or a long-term problem of winning the goal, making the likelihood of punting less continuously updated than would be rational (Sobrepere et al., 2022). Soccer players fouling after losing the ball similarly suggest switching the construal from normal team play to personal retaliation, with the risk to the team from fouling no longer affecting the foul decision if the player can foul the opposing player who had stripped him of the ball (Greve et al., 2019a). Again, the change away from decision-making based on team goals is obvious because greater probability of a referee calling a foul nearly always reduces the likelihood of fouling. This change resembles the evidence on individual decision-makers turning to self-enhancement when performance on goals suggests a need for problemistic search (Jordan and Audia, 2012; Audia et al., 2015).

## Implications for psychology research

It follows from this review that the mismatch between much psychological research on individual goals and Carnegie School research on organizational goals is even greater than at first glance. We know much about personal goal selection and pursuit, but organizations assign goals to individuals and require or incentivize their pursuit. We know much about how broad and high-level goals trigger various organizational changes, but these changes are preceded by construal processes that determine whether the individual believes that change is needed and if so, what type of change. The microfoundation of current Carnegie School research owes more to observation of organizational decision-making than to psychological research.

Fortunately, the questions that currently most urgently require answers in the Carnegie School play to central strengths of social psychology. Organizational decision-makers are boundedly rational, and apply construal to accurately understand the situation while maintaining a sense of self-worth (e.g., Kunda, 1990). Applying this to Carnegie School research implies a closer look at how individuals process information on goals and performance, along with information on what actions are available, to form construals and make decisions. Inspiration for this research can be found in field research on decisions made by organizations (Clough and Piezunka, 2020; Lim and Audia, 2020; Hu et al., 2022), mostly with decision-makers and processes not observed by the researcher, along with analysis from areas such as sports (Raab et al., 2012; Greve et al., 2019a; Sobrepere et al., 2022), with superior documentation of who decides what, but still without experimental control. Social psychology has a research stream devoted to construal processes, and its experimental method is the most efficient causally oriented method for understanding them.

### Satisficing

Each of the unanswered questions of the Carnegie School corresponds to existing or potential social psychological studies. First, what does satisficing mean? The Carnegie School views failure to meet goals as an interruption mechanism that triggers consideration of whether to initiate search. The role of overriding goals such as profitability and more focused goals (market share, safety, growth, customer satisfaction, and so on) relative to each other in triggering search and change needs additional empirical investigation from a construal perspective. For example, recent work shows that construal of low profitability either as a problem shared across firms or as one unique to the focal firm influences responses (Lucas et al., 2018; Goyal and Goyal, 2021). Also, self-enhancement research has documented that multiple goals or aspiration levels open for multiple forms of construal, and can lead to inaction in the face of performance levels that are low enough to indicate that problemistic search is needed (Audia and Brion, 2007; Audia and Greve, 2021). Recent research has documented that this relation is moderated by greater success or higher status, which gives sufficient confidence to reduce self-enhancement (Kostopoulos et al., 2022). Personal characteristics also matter, with overconfident CEOs being less likely to view low performance as a sign that the firm needs to change (Schumacher et al., 2020), possibly because they interpret ambiguous situations favorably and hence persist with current behaviors (Halper and Vancouver, 2016).

The research also needs better connection with research on how self-efficacy influences change, especially because there is a current debate on whether self-efficacy effects on confidence and effort add up to increased or decreased performance (Audia et al., 2000; Vancouver et al., 2002; Schmidt and DeShon, 2010). These studies and their potential relation to CEO experience give good reason to examine self-efficacy in organizations further (Tarakci et al., 2018). Status, success, confidence, and self-efficacy are a complex blend of similar characteristics with effects that appear to be in partial contrast to each other, and further work is needed to sort them out.

### Numeric control

Second, how are aspiration levels updated and interpreted? The use of aspiration levels to assess whether there are shortfalls is so important for the individuals responsible for the goals that it is clearly a case of motivated inference influenced by a wish to correctly understand the situation, but also to maintain a positive self-assessment. It is not well known how organizations explicitly stated numeric targets affects this process because most research so far has ignored numeric targets, and instead tracked adaptive aspiration levels. Experimental evidence on updating aspiration levels is available (Lant, 1992), but recent work on how aspiration levels are updated in organizations has suggested more mechanisms that require additional research, with greater emphasis on experiments than most current research (Bromiley and Harris, 2014; Moliterno et al., 2014; Blettner et al., 2015; Kacperczyk et al., 2015). Importantly, although the effects of organizational updating of aspiration levels are well-documented and regular, individual updating and response to aspiration levels is more heterogeneous (Banerjee et al., 2019; Bergenholtz et al., 2023). To transition from a performance shortfall to a search decision, individuals need to infer a meaning from the performance shortfall.

Another unexplored question is whether decision-makers switch from the “magic numbers” assessment of numerically specified goals to a broader goal conception when the performance is below the aspiration level, thus producing either a more informed decision—or another reason for self-enhancement. Here, a useful observation is that ambiguity complicates the search for meaning (Plambeck and Weber, 2010; Joseph and Gaba, 2015), so before the firm can search for solutions it may collect additional information that helps classify the problem it is facing (Glynn et al., 2019). Whether such information collection occurs also involves construal because it involves the perceived urgency of a resolution (Liberman and Trope, 1998). Broader goal conceptions also matter because the organizational environment increasingly includes external goals such as those on environmental, social, and governance dimensions. External goals can intervene at unpredictable times (Kölbel et al., 2017), complicating the decision-making. More work on how ambiguous performance feedback triggers search for meaning is needed.

### Directing search

Third, how is search directed? Search direction implies selecting a specific goal shortfall and pursuing its resolution. The organizational decision-maker faces information on shortfalls in one or multiple goals, and these goals differ in importance and in specificity for attributing the reason for the shortfall and its potential solution. Again, construal processes are central, with attributions of events taking a central role, perhaps combined with heuristics (Gigerenzer and Gaissmaier, 2011; Wilson, 2022). Work so far has yielded promising findings on how search is directed. For example, similar profitability shortfalls can be construed as excess or insufficient resources depending on the overall resource endowment of the organization (Kuusela et al., 2017). Similar levels of failure in corporate acquisitions are less likely to lead to divestment when they can only be construed as the responsibility of the current CEO (Hayward and Shimizu, 2006). Sports teams pursue goals of revenue and status, and direct search toward recruitment of famous, versus effective, players depending on which goal sees shortfalls (Ertug and Castellucci, 2013).

Research has also shown that distant search is not only initiated after proximate search fails (e.g., Iyer et al., 2019), as predicted, but also by severe performance shortfalls (Billinger et al., 2021). Shortfalls in organizational goals are particularly consequential for managers identifying strongly with the organization (Tarakci et al., 2018). Also, difficulty in construal due to perceived ambiguity leads to more distant search and greater variety of organizational changes (Plambeck and Weber, 2009). Clearly, decisions to make radical departures from current behaviors are motivated by difficult circumstances. These findings demonstrate the power of the situation in directing search, with significant consequences for organizations. Currently there are so many interesting ideas and unresolved questions that understanding the process of directing search and its underlying construal requires a significant investment in experimental research.

When examining how search is directed, it is important to keep in mind an important lesson from the field research of the Carnegie School: Directing search is also influenced by the organizational past. Organizational learning directs search by giving information helpful for forming construal and determining response (Argote and Greve, 2007). In the long run, organizational responses are learned and retained through processes such as storytelling (Myers, 2022), routine formation (Bresman, 2013), and job creation (Miner, 1990). In the short run, information from the environment shapes construal processes and influences the response. Organizational networks influence organizational actions directly (Brass et al., 2004) or through modifying the salience of alternatives considered when initiating search (Hu et al., 2022). These learning processes are well-documented and can be a source of ideas when designing experiments (Gavetti et al., 2012).

## Conclusion

It is central to Carnegie School research that goals control the behaviors of the organization and its members. They do so by structuring the situation facing each decision-maker. Goals and the associated aspiration levels define what is important, how it is measured, what performance level is satisfactory, when to look for alternative behaviors, and what alternatives are good enough. This is a very high degree of situational control obtained through a simple metric. The Carnegie School has produced abundant research showing the practical consequences of goals, and the effects have significant magnitude in most studies.

Now would be a good time to see research that lays out the psychological processes that underlie these effects. Is there a straightforward connection between the evidence on goals that individuals choose for themselves and goals that organizations impose on them? Do we understand the difference between situations that compel the decision-maker to engage in problem-solving behaviors and situations that allow self-enhancement or construal that permit inaction? Can we tell how goals and other situational factors influence construal so that search is initiated—and directed? Scholarship on organizational responses has been quite person-less for a long time and has made significant progress even so. Imagine how much further research on organizational goals could go if the insights from the Carnegie School were used as a starting point for additional research to fill the gaps outlined in this paper. We would understand the underlying micro-processes and we would be better positioned to explain how search is directed and suitable solutions are found.

## Author contributions

The author confirms being the sole contributor of this work and has approved it for publication.

## Conflict of interest

The author declares that the research was conducted in the absence of any commercial or financial relationships that could be construed as a potential conflict of interest.

The handling editor, PA, declared a past co-authorship with the author, HG.

## Publisher’s note

All claims expressed in this article are solely those of the authors and do not necessarily represent those of their affiliated organizations, or those of the publisher, the editors and the reviewers. Any product that may be evaluated in this article, or claim that may be made by its manufacturer, is not guaranteed or endorsed by the publisher.

## Footnotes

^1^In most management research, the term "interpretation" is used instead of construal. The meanings are slightly different, but the common implication that interpretation is consciously done is not central to how it is used theoretically, so construal can be substituted.
